# Ecological data in Darwin Core: the case of earthworm surveys

**DOI:** 10.3897/BDJ.9.e71292

**Published:** 2021-12-08

**Authors:** Maxim Shashkov, Natalya Ivanova, John Wieczorek

**Affiliations:** 1 Institute of Physicochemical and Biological Problems in Soil Science of the Russian Academy of Sciences (RAS), Pushchino, Russia Institute of Physicochemical and Biological Problems in Soil Science of the Russian Academy of Sciences (RAS) Pushchino Russia; 2 Institute of Mathematical Problems of Biology RAS – the Branch of Keldysh Institute of Applied Mathematics of Russian Academy of Sciences, Pushchino, Russia Institute of Mathematical Problems of Biology RAS – the Branch of Keldysh Institute of Applied Mathematics of Russian Academy of Sciences Pushchino Russia; 3 VertNet, Bariloche, Argentina VertNet Bariloche Argentina; 4 University of California, Berkeley, United States of America University of California Berkeley United States of America

**Keywords:** sampling-event data, soil biodiversity, "Kaluzhskiye Zaseki" Nature Reserve, Ugra National Park

## Abstract

**Background:**

This sampling-event dataset provides primary data about species diversity, age structure, abundance (in terms of biomass and density) and seasonal activity of earthworms (Lumbricidae). The study was carried out in old-growth broad-leaved and young forests of two protected areas ("Kaluzhskiye Zaseki" Nature Reserve and Ugra National Park) of Kaluga Oblast (Russia).

**New information:**

The published dataset provides new data about earthworm communities in European Russia. We propose a new schema according to Darwin Core for the standardisation of the soil invertebrates survey data.

## Introduction

Earthworms occur in soils almost across the whole world, preferring moist habitats of moderate temperature. They are amongst the major components of terrestrial ecosystems dominating the biomass of soil invertebrates in non-acidic soils (Lee 1985, Edwards and Bohlen 1996). Through burrowing, casting and mixing of litter and soil (bioturbation), they influence aggregate stability, soil structure, infiltration of water, aeration of deeper soil layers, nutrient cycling, microbial biomass and other soil invertebrates (Eisenhauer et al. 2007, Eisenhauer 2010), and so linked with the development of sustainable forest ecosystems (Lavelle et al. 1997, Blouin et al. 2013). Despite this, the amount of available data on the distribution of earthworms in the world is very limited. Recent studies (Cameron 2018, Phillips 2019, Phillips 2021) have highlighted many gaps in our knowledge of the distribution of Lumbricidae, amongst which the territory of Russia is characterised by an extremely low amount of available data. For example, GBIF.org provides only 9602 occurrences of family Lumbricidae for the Russian territory (GBIF.org. 2021) in contrast to an extensive scientific heritage accumulated by Soviet and Russian researchers (Baluev 1950, Malevich 1954, Horizonova et al. 1957, Malevich and Perel 1958, Malevich 1959, Vsevolodova-Perel 1997, Striganova and Porjadina 2005, Berman et al. 2009, Makarova and Kolesnikova 2019, Shekhovtsov et al. 2020 and many others).

In our opinion, this situation can be explained by two reasons. The first one is time-consuming and labour-intensive field data collection (Coja et al. 2008), which does not allow continuous gathering of material from many locations. There are different methods used for earthworm extraction from the soil. The most widely used technique for quantitative sampling of earthworms is hand-digging and hand-sorting (Satchel 1969, Edwards and Lofty 1977, Lee 1985), as well as a formalin extraction method (Raw 1959), electrical octet method (Rushton and Luff 1984, Bohlen et al. 1995, Eisenhauer et al. 2008), hot mustard (Gunn 1992, Eisenhauer et al. 2008) and onion extraction (Steffen et al. 2013) methods.

The matter that the data standardisation process is not clear is the second barrier to earthworm data exchange and integration because, usually, earthworms are collected according to sampling-event design. Nowadays, Darwin Core (Wieczorek et al. 2012) is the key data standard for biodiversity data mobilisation through the GBIF portal. This specimen-based standard was developed for describing species point records and has served as the basis for the interoperability of taxonomic and occurrence-based datasets. However, it has its origin in the natural history collections community and was not initially intended to capture metadata about multi-species sampling processes (Wiser et al. 2011, Guralnick et al. 2018). Although recent efforts have begun to develop an ‘Event Core’, as well as new terms that related to ecological data mobilisation (dwc: samplingProtocol, dwc: sampleSizeValue, dwc: sampleSizeUnit, dwc: samplingEffort), the contribution of sampling-event datasets to GBIF remains low (3.1% of all published datasets). The Humboldt Core TDWG task group is working to develop a new standard for biodiversity inventory data sharing. However, ecological data, as well as data collection protocols, are often so different, even for studying the same taxonomic group. For example, species data in earthworms censuses may be available for the whole census, each soil sample in the survey or each soil sample layer in the soil sample. In this case, it is not always clear what an event is. At the same time, it is essential to establish the possibility of combining surveys from different datasets.

Here, we provide the sampling event dataset of long-term earthworm surveys (Shashkov and Ivanova 2021), carried out in protected areas of Kaluga Oblast (Russia) (Shashkov 2014, Shashkov 2016), with detailed data on species in soil sample layers, as well as the schema for representing this data in the Darwin Core.

## General description

### Purpose

1. Provide high-quality soil biodiversity data.
2. Suggest the schema for earthworms surveys data standardisation according to Darwin Core.

### Additional information

**Figure 1.**
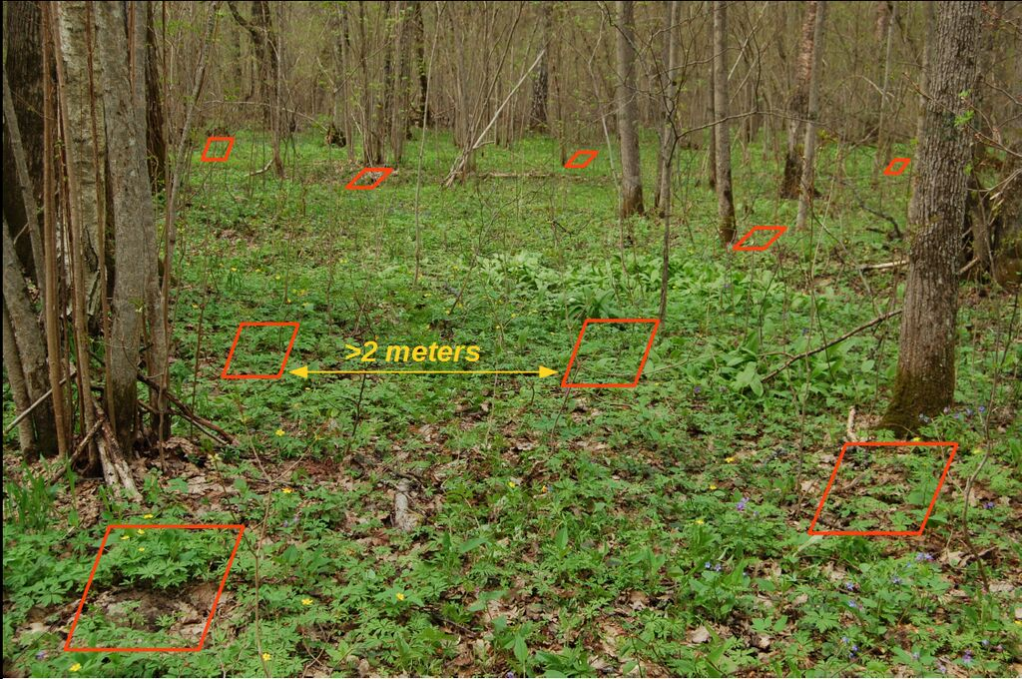
Soil sample locations on the study site.

We used data collected by the hand-sorting method in our example. During each survey (usually taken during one day), soil samples of fixed size were randomly collected within the sampling plot (in similar tree and herb cover and soil type). Each soil monolith was hand-sorted by layers for earthworms (see details in the Sampling description section). An example of sampling design is shown in Fig. 1. Geographic coordinates were recorded for the sampling plot, not for each soil sample. Some sampling plots were studied once, others - several times during the year or several years.

**Figure 2.**
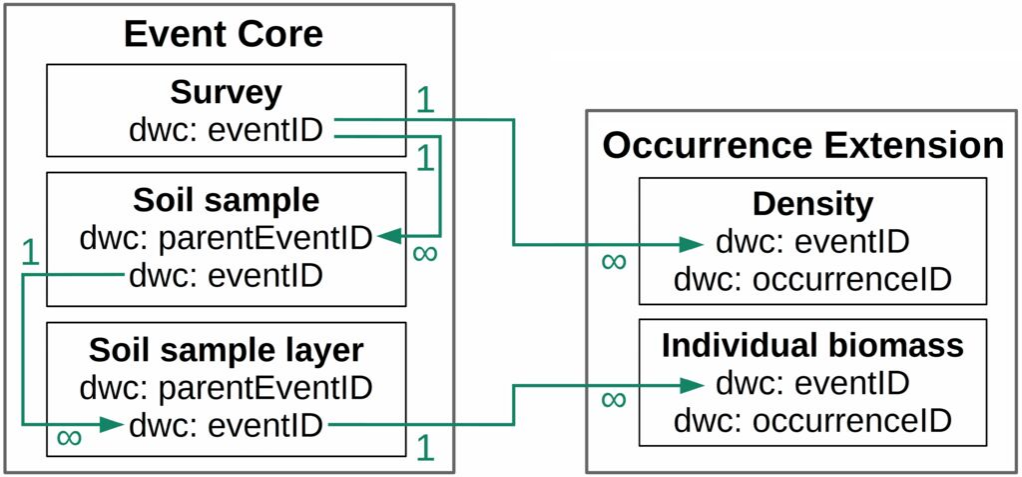
Event hierarchy of long-term earthworms surveys.

Thus, our primary data included information for each individual in the soil sample (species, biomass and life stage) and earthworm density (number of individuals) for the survey. During the data standardisation process, we considered three types of events (Fig. 2), connected hierarchically. The most large-scale event is a survey. One survey is a set of soil samples collected at one location during one sampling period. The second level is a soil sample. It is a part of the survey, each soil sample collected during the survey, including empty samples. The third level is a soil sample layer. It is a part of the soil sample.

Thus, we included in the dataset occurrences of two levels (Table 1): individual specimens occurrences assigned to soil sample layer (with individual biomass and life stage) and occurrences assigned to a survey (with total density).

Used event hierarchy allowed us to maintain data consistency and completeness. Nevertheless, our method has some bottlenecks. Firstly, it is not common practice to combine events of different levels in one dataset. At the same time, each event level should be described in the dataset. This information requires a particular Darwin Core term, but it is currently absent. We used the general term dwc: dynamicProperties as a temporary solution in this work. Secondly, the event hierarchy includes 338 events (the soil sample level), which are not assigned to any occurrences. These events are empty not because no species were registered. We used this event level for the relationship between survey and sample layer event types. However, empty events are not shown on the GBIF dataset page. Moreover, complete data (with empty events) are available for download via the IPT installation page, not the GBIF interface. This fact restricts the reuse of our data.

Possibly, another data standardisation design could be more understandable. It would be simpler to use the soil sample as the event and bind samples from one sample plot via dwc: locationID and different surveys via dwc: parentEventID. This scheme avoids empty events not related to occurrences. However, its implementation is not possible due to technical IPT limitations. We cannot assign different depths for occurrences into one event because dwc: verbatimDepth, dwc: minimumDepthInMeters and dwc: maximumDepthInMeters are related to the Event Core.

On the other hand, events of different levels made it possible to provide different level traits. In our dataset, we provided life stage and biomass for each specimen and density for the survey. This is an essential advantage for ecological data re-analysis.

Overall, our solution is not optimal. This approach is a trade-off between the need to provide as complete data as possible, the current state of the Darwin Core standard and the technical limitations of the IPT. We believe that further development of biodiversity data standards and data publishing protocols will optimise the process of ecological sampling-event data mobilisation and facilitate their reuse.

## Sampling methods

### Study extent

**Figure 3.**
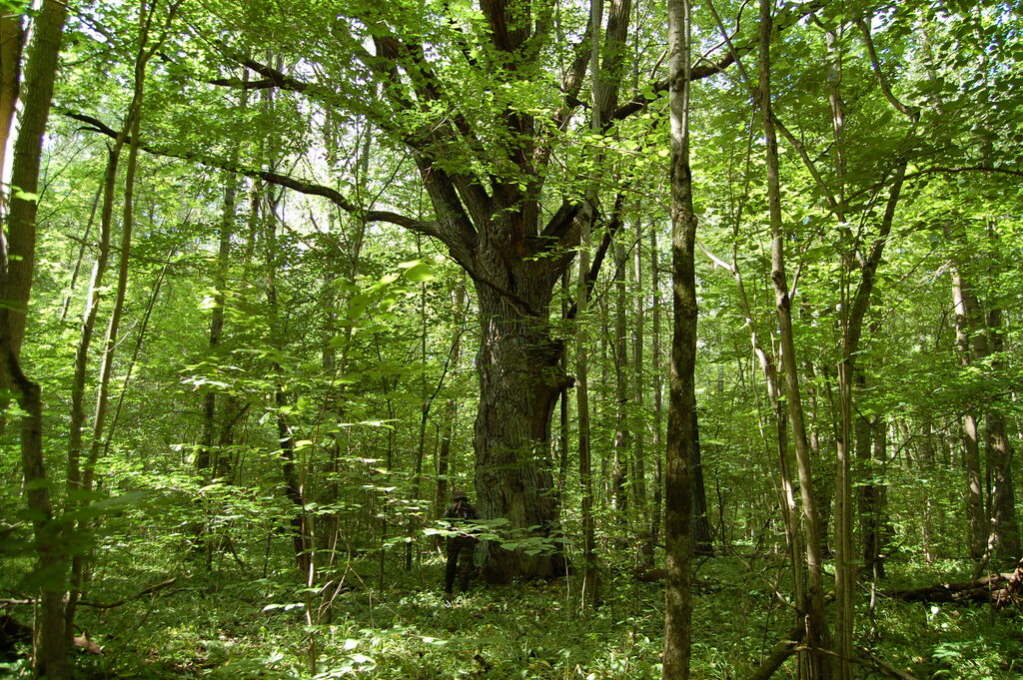
The old-growth broad-leaved subclimax forest site.

**Figure 4.**
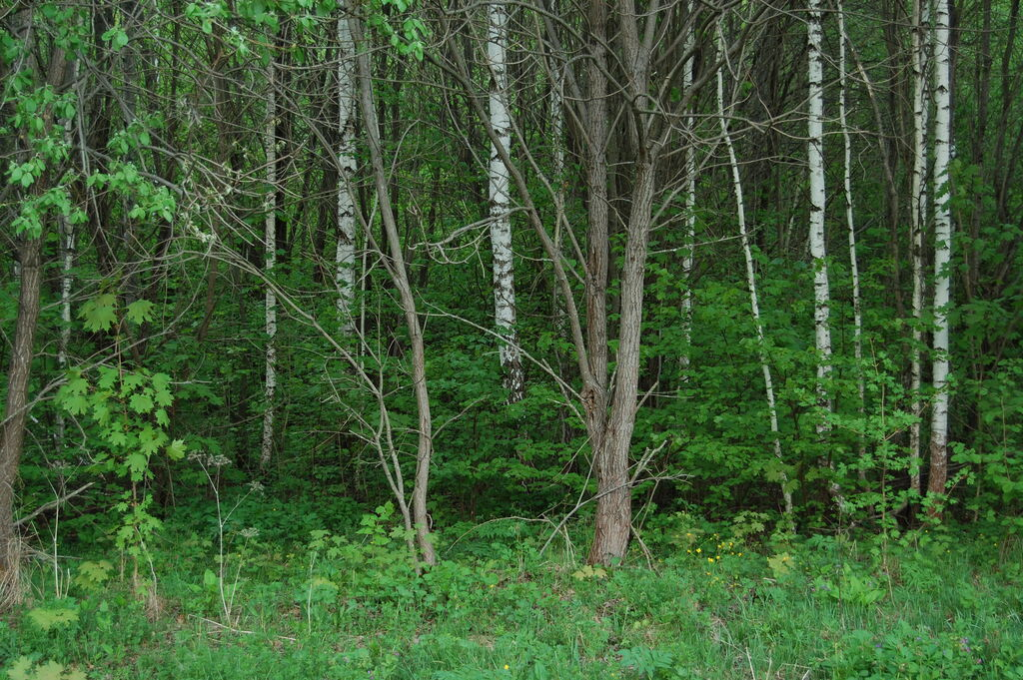
Young forest site with stand of birch and willow on the former agricultural land.

The study area was located in the central part of the East European Plain. Earthworms were collected in 13 locations of old-growth broad-leaved forests and young birch forests in the "Kaluzhskiye Zaseki" Nature Reserve and Ugra National Park. There were 10 sampling plots in old-growth broad-leaved forests at a late successional stage or subclimax (Fig. 3). All of them, but one (Val), were located either on the watershed or watershed slope. Two more sites in 30-year birch forests with broad-leaves regrowth at an early stage of reforestation succession (Fig. 4), one in a locality of former tillage and the second one in a locality of former pasture, were sampled. One more sample plot represented black alder forest in the floodplain (Table 2).

The old-growth forest stands consist of *Quercusrobur* L., *Fraxinusexcelsior* L., *Tiliacordata* Mill., *Ulmusglabra* Huds., *Acerplatanoides* L., *Acercampestre* L., *Betula* spp. and *Populustremula* L. with regrowth of the broad-leaved tree species, except for oak. The herbal layer is dominated by *Aegopodiumpodagraria* L., *Mercurialisperennis* L., *Galeobdolonluteum* Huds., *Pulmonariaobscura* Dumort. and nitrophilous fern *Matteucciastruthiopteris* (L.) Tod.

The second investigated group of forest stands comprises young forests established on abandoned arable field and pasture. The stands of young forest are predominantly composed of *Betula* spp. and *Salixcaprea* L. Sampling plots were located on abandoned farmlands. The distance to the edgeof old-growth forests was about 30-50 metres.

### Sampling description

**Figure 5.**
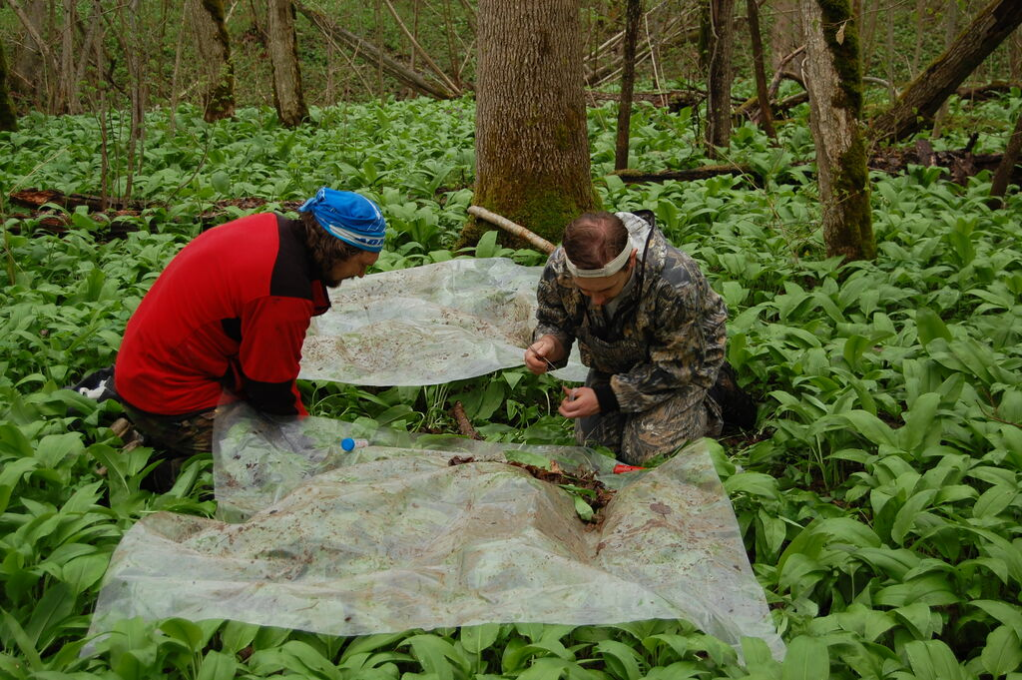
Earthworms hand-sorting in old-growth forest site.

**Figure 6.**
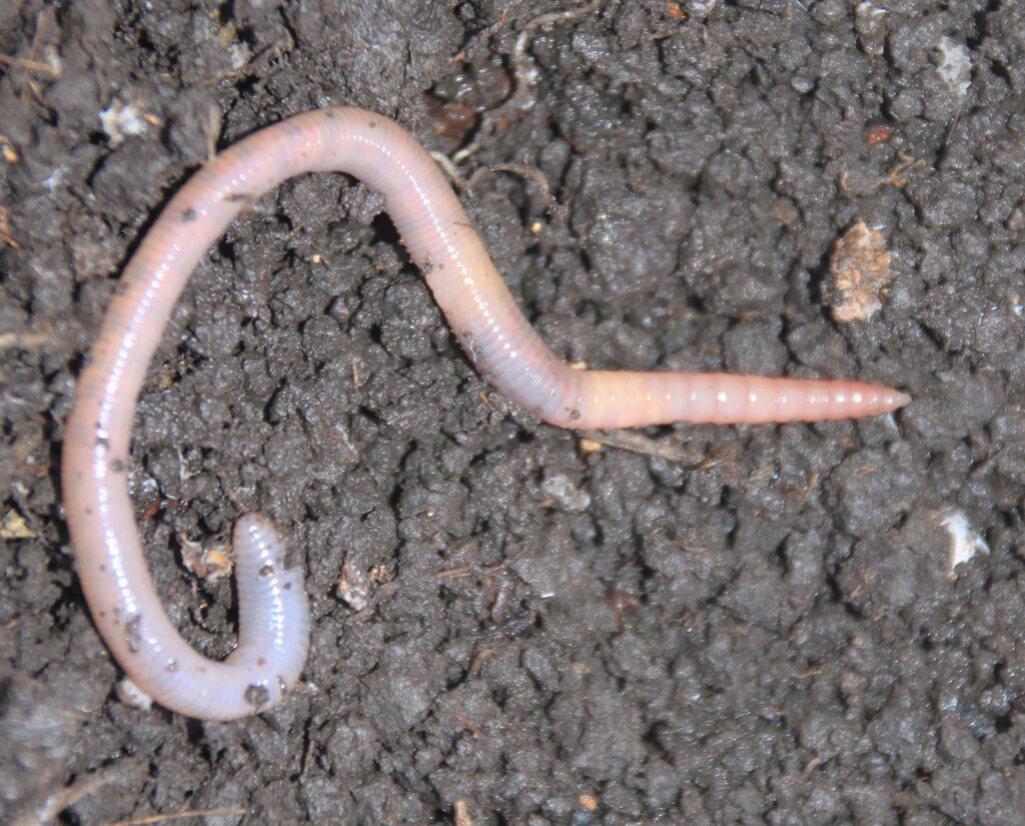
Specimen of the most abundant species - *Aporrectodeacaliginosa*. Subadult ontogenetic stage. The photo was taken on 28 March 2016 by Maxim Shashkov on sampling plot R4. Then soil was covered by packed snow and crust of 30-40 depth. The worm was active in the topsoil layer just under the snow.

At each sampling plot, 8-24 randomly located soil samples (25 cm × 25 cm) were dug to a depth of 35 cm for earthworms collection (Ghilarov 1975). Soil monoliths were taken, if possible, under the middle of the crown projection of a large tree between the crown edge and the trunk, for reducing the possible influence of microstational condition differences. Earthworms were separated from soil by hand-sorting onsite (Fig. 5 and Fig. 6) by layers: litter (A0), 0-10 cm, 10-20 cm and >20 cm. Collected earthworm specimens were preserved in 4% formaldehyde, transferred to the laboratory and, if possible, identified to species level. Specimens were identified using the key of Vsevolodova-Perel (1997) by Maxim Shashkov. Most of the juvenile specimens were identified to species level, except ones belonging to the genus *Lumbricus*. Identification of some specimens was confirmed by T.S. Vsevolodova-Perel personally.

## Geographic coverage

### Description

Kaluga Oblast, Russian Federation

### Coordinates

53.615 and 53.922 Latitude; 35.732 and 35.881 Longitude.

## Taxonomic coverage

### Taxa included

**Table:** 

Rank	Scientific Name	
family	Lumbricidae	
species	*Octolasionlacteum* Örley, 1881	
genus	*Aporrectodea* Orley, 1885	
species	*Aporrectodearosea* (Savigny, 1826)	
species	*Aporrectodeacaliginosa* (Savigny, 1826)	
genus	*Lumbricus* Linnaeus, 1758	
species	*Lumbricusterrestris* Linnaeus, 1758	
species	*Lumbricusrubellus* Hoffmeister, 1843	
species	*Lumbricuscastaneus* (Savigny, 1826)	
species	*Eisenianordenskioldi* (Eisen, 1879)	
species	*Dendrobaenaoctaedra* (Savigny, 1826)	

## Traits coverage

The dataset provides three trait types.

### Life stage

Earthworms were distinguished into three ontogenetic stages – juvenile, subadult and adult, based on the development of the clitellum. It is the reproductive gland used for cocoon production by mature earthworms generally forming an obvious band around the mid-section segments. Adult earthworms had a fully developed clitellum. Earthworms were considered subadult if they had any signs of tubercula pubertatis, but no clitellum and adult if they are clitellate (Sims and Gerard 1999). Earthworms were considered juveniles if they had neither tubercula pubertatis nor clitellum. Cocoons were not taken, as the washing method is more suitable for cocoons collection, but takes more time than hand-sorting (Singh et al. 2015). Occasionally, found cocoons were not included in the dataset because of the impossibility of identifying them by morphological features.

### Biomass

Preserved specimens were weighed to determine earthworm biomass with portative balance Ohaus SPU 123. This device allows taking weight with precision of 0.001 g with an accuracy of 0.003 g. All the worms were weighed under laboratory conditions in a preserved state. No corrections were made for gut content or dehydration in formaldehyde. Individual biomass was in the range of 2 to 5220 mg. The largest worms were specimens of *Aporrectodeacaliginosa* (max. 1630 mg) and *Lumbricusterrestris*. The total biomass was highest in old-growth forests on Phaozems (61.4-110.5 g/m^2^) and Luvisols grey (45.9-104.0 g/m^2^), as well as the young forest on former pasture (97.3-135.9 g/m^2^). The lowest values were recorded for the young forest on former arable land (4.4-43.5 g/m^2^) and the alder forest experiencing seasonal flooding (17.9-25.1 g/m^2^).

### Density

Some worms were damaged during soil excavation with a shovel. The fragment was considered a specimen when it had an anterior end, but each counted for biomass. The most abundant population of earthworms in terms of relative density (individuals per square metre) was revealed in the old-growth forest on Phaozem (R1) and in the young forest on the former pasture. The poorest values were observed in the young forests on the former arable soil.

## Temporal coverage

**Data range:** 2000-8-20 – 2012-9-25.

### Notes

See Table 2 for details.

## Usage licence

### Usage licence

Other

## Data resources

### Data package title

Earthworm communities (Oligochaeta: Lumbricidae) in old-growth and young forests of protected areas of the Kaluga Oblast (European Russia).

### Resource link

https://www.gbif.org/dataset/f6822eb1-b570-4566-98b0-894d4213510e

### Number of data sets

1

### Data set 1.

#### Data set name

Earthworm communities (Oligochaeta: Lumbricidae) in old-growth and young forests of protected areas of the Kaluga Oblast (European Russia).

#### Data format

Darwin Core archive

#### Number of columns

36

#### Character set

UTF-8

#### Download URL

http://gbif.ru:8080/ipt/archive.do?r=worms_survey

**Data set 1.** 

Column label	Column description
eventID(Darwin Core Event, Darwin Core Occurrence Extension)	An identifier for the set of information associated with an Event (survey, soil sample or soil sample layer). https://dwc.tdwg.org/terms/#dwc:eventID 1005 unique values, examples: "R5:2012-09:3", "R5:2012-09:6:3:>10".
parentEventID(Darwin Core Event)	An identifier for the broader Event that groups this and potentially other Events (survey or soil sample). https://dwc.tdwg.org/terms/#dwc:parentEventID 372 unique values, examples: "R3:2006-05", "P2:VZv:2003-05:7".
dynamicProperties(Darwin Core Event)	Description of the Event in JSON format. https://dwc.tdwg.org/terms/#dwc:dynamicProperties Example: "{'event type':'soil sample','part of survey':'R1:2012-06'}".
eventDate(Darwin Core Event)	The date which an Event occurred (YYYY-MM-DD format). https://dwc.tdwg.org/terms/#dwc:eventDate 22 unique values ranged between '2000-08-20' and '2012-09-25'.
samplingProtocol(Darwin Core Event)	The description of the method used during an Event. https://dwc.tdwg.org/terms/#dwc:samplingProtocol Constant: "Digging-out and hand-sorting (by layers) of the soil samples of 25 * 25 cm and a depth of ca. 35 cm".
sampleSizeValue(Darwin Core Event)	A numeric value for a measurement of the size of a sample in a sampling event (number of soil samples for the 'plot survey' event, size of the soil sample for the 'soil sample' event and area of sampling for the 'soil sample layer' event). https://dwc.tdwg.org/terms/#dwc:sampleSizeValue Constant for soil and layer level: "25×25×35" and "0.0625", respectively.
sampleSizeUnit(Darwin Core Event)	The unit of measurement of the size of a sample in a sampling event. https://dwc.tdwg.org/terms/#dwc:sampleSizeUnit Constant for each level: "soil samples", "centimetres" and "square centimetres" - survey, soil sample and layer, respectively.
locationID(Darwin Core Event)	An identifier for the sampling plot. https://dwc.tdwg.org/terms/#dwc:locationID 13 unique values, examples: "R2", "VZv", "33kv".
countryCode(Darwin Core Event)	The standard code for the country in which the Location occurs according to ISO 3166-1-alpha-2. https://dwc.tdwg.org/terms/#dwc:countryCode Constant: "RU".
country(Darwin Core Event)	The name of the country or major administrative unit in which the Location occurs. https://dwc.tdwg.org/terms/#dwc:county Constant: "Russian Federation".
stateProvince(Darwin Core Event)	The name of the next smaller administrative region than country in which the Location occurs. https://dwc.tdwg.org/terms/#dwc:stateProvince Constant: "Kaluga Oblast".
locality(Darwin Core Event)	Protected area name. Three possible values: "Ugra National Park" , "Kaluzhskiye Zaseki Nature Reserve (Southern cluster)" or "Kaluzhskiye Zaseki Nature Reserve (Northern cluster)". https://dwc.tdwg.org/terms/#dwc:locality
decimalLatitude(Darwin Core Event)	The geographic latitude (in decimal degrees, using the spatial reference system given in geodeticDatum) of the geographic centre of a Location. https://dwc.tdwg.org/terms/#dwc:decimalLatitude Ranged berween: 53.6148 and 53.92215.
decimalLongitude(Darwin Core Event)	The geographic longitude (in decimal degrees, using the spatial reference system given in geodeticDatum) of the geographic cenere of a Location. https://dwc.tdwg.org/terms/#dwc:decimalLongitude Ranged between: 35.73175 and 35.88146.
geodeticDatum(Darwin Core Event)	The ellipsoid, geodetic datum or spatial reference system (SRS) upon which the geographic coordinates given in decimalLatitude and decimalLongitude are based. https://dwc.tdwg.org/terms/#dwc:geodeticDatum Constant: "WGS84".
coordinateUncertaintyInMeters (Darwin Core Event)	The horizontal distance (in metres) from the given decimalLatitude and decimalLongitude describing the smallest circle containing the whole of the Location. https://dwc.tdwg.org/terms/#dwc:coordinateUncertaintyInMeters Constant: 50.
coordinatePrecision(Darwin Core Event)	A decimal representation of the precision of the coordinates given in the decimalLatitude and decimalLongitude. https://dwc.tdwg.org/terms/#dwc:coordinatePrecision Constant: 0.00001.
minimumDepthInMeters(Darwin Core Event)	The lesser depth of a range of depth below the local surface, in metres. https://dwc.tdwg.org/terms/#dwc:minimumDepthInMeters Values: 0.0, -0.1, -0.2.
maximumDepthInMeters(Darwin Core Event)	The greater depth of a range of depth below the local surface, in metres. https://dwc.tdwg.org/terms/#dwc:maximumDepthInMeters Values: 0.0 (litter considered above 0), -0.1, -0.2, -0.35.
habitat (Darwin Core Event)	A description of the habitat in which the Event occurred.https://dwc.tdwg.org/terms/#dwc:habitat 5 unique values, examples: "Broad-leaved forest", "Young birch forest".
occurrenceID(Darwin Core Occurrence Extension)	An identifier for the Occurrence. https://dwc.tdwg.org/terms/#dwc:occurrenceID 6935 unique values, example: "758-P2:VZv:2003-09:5:2:0-10".
basisOfRecord(Darwin Core Occurrence Extension)	The specific nature of the data record. https://dwc.tdwg.org/terms/#dwc:basisOfRecord Constant: "PreservedSpecimen".
occurrenceStatus(Darwin Core Occurrence Extension)	A statement about the presence or absence of a Taxon at a Location. https://dwc.tdwg.org/terms/#dwc:occurrenceStatus Constant: "present".
scientificName(Darwin Core Occurrence Extension)	The full scientific name according GBIF Backbone checklist. https://dwc.tdwg.org/terms/#dwc:scientificName 11 unique values, example: "Lumbricus Linnaeus, 1758", "Eisenianordenskioldi (Eisen, 1879)".
kingdom (Darwin Core Occurrence Extension)	The full scientific name of the kingdom in which the taxon is classified. https://dwc.tdwg.org/terms/#dwc:kingdom Constant: "Animalia".
taxonRank(Darwin Core Occurrence Extension)	The taxonomic rank of the most specific name in the scientificName. https://dwc.tdwg.org/terms/#dwc:taxonRank Values: "FAMILY", "GENUS", "SPECIES".
identificationReferences(Darwin Core Occurrence Extension)	Source of reference used in the Identification. https://dwc.tdwg.org/terms/#dwc:identificationReferences Constant: "Vsevolodova-Perel T.S. The earthworms of the fauna of Russia ...".
lifeStage(Darwin Core Occurrence Extension)	The life stage of the biological individual at the time the Occurrence was recorded. https://dwc.tdwg.org/terms/#dwc:lifeStage Possible values: "Juvenile", "Subadult", "Adult".
individualCount(Darwin Core Occurrence Extension)	The number of individuals represented present at the time of the Occurrence (was counted for 'survey' event). https://dwc.tdwg.org/terms/#dwc:individualCount Ranged between 1 and 260.
organismQuantity(Darwin Core Occurrence Extension)	A value for the quantity of organisms, depends on unit (Quantity Type). https://dwc.tdwg.org/terms/#dwc:organismQuantity
organismQuantityType(Darwin Core Occurrence Extension)	The type of quantification system used for the quantity of organisms. https://dwc.tdwg.org/terms/#dwc:organismQuantityType Two possible values: "gram" and "individuals/per survey".
recordedBy(Darwin Core Occurrence Extension)	A person responsible for recording the original Occurrence. https://dwc.tdwg.org/terms/#dwc:recordedBy Constant: "Maxim Shashkov".
institutionID(Darwin Core Occurrence Extension)	An identifier for the institution having custody of information referred to in the record (https://issp.pbcras.ru/). https://dwc.tdwg.org/terms/#dwc:institutionID Constant: "https://issp.pbcras.ru/".
institutionCode(Darwin Core Occurrence Extension)	The name of the institution having custody of information referred to in the record. https://dwc.tdwg.org/terms/#dwc:institutionCode Constant: "Institute of Physicochemical and Biological Problems in Soil Science of the Russian Academy of Sciences".
ownerInstitutionCode(Darwin Core Occurrence Extension)	The name of the institution having ownership of information referred to in the record (Pushchino Scientific Center for Biological Research of the Russian Academy of Sciences). https://dwc.tdwg.org/terms/#dwc:ownerInstitutionCode Constant: "Pushchino Scientific Center for Biological Research of the Russian Academy of Sciences".
identifiedBy(Darwin Core Occurrence Extension)	The person, who assigned the Taxon to thesubject. https://dwc.tdwg.org/terms/#dwc:identifiedBy Constant: "Maxim Shashkov".

## Figures and Tables

**Table 1.** Events types, associated occurrences and traits.

Event type	Number of events	Number of associated occurrences	Traits
The survey	39	271	Density
Soil sample	338	0	-
Soil sample layer	628	6673	Individual biomass, life stage

**Table 2.** Main characteristics of earthworm survey plots and temporal coverage.

Sampling plot code (dwc: locationID)	Protected area (dwc: locality)	Survey periods	Coordinates	Habitat (dwc: habitat)	Soil type
T1	Ugra National Park	May, June and September 2003, June 2004	N 53.89400, E 35.86468	Broad-leaved forest	Luvisol grey forest
T2	Ugra National Park	May, June and September 2003, June 2004	N 53.90408, E 35.83320	Broad-leaved forest	Luvisol grey forest slightly podzolics
VZv	Ugra National Park	May, June and September 2003, June 2004	N 53.88742, E 35.81388	Broad-leaved forest	Luvisol light grey forest
Poima	Ugra National Park	September 2003, June 2004	N 53.92215, E 35.73175	Black alder forest, small river floodplain	Luvisol alluvial gleic
Val	Ugra National Park	May, June 2003, June 2004	N 53.91861, E 35.73266	Broad-leaved forest, natural levee of oxbow	Luvisol illuvial-ferruginous
33 kv	Kaluzhskiye Zaseki Nature Reserve (Northern cluster)	August 2004	N 53.77853, E 35.73524	Broad-leaved forest	Luvisol sod illuvial-ferruginous contact-gleyic
43 kv	Kaluzhskiye Zaseki Nature Reserve (Northern cluster)	August 2004	N 53.76148, E 35.73751	Broad-leaved forest	Luvisol sod illuvial-ferruginous
R1	Kaluzhskiye Zaseki Nature Reserve (Southern cluster)	May 2006, July 2011, May, June, September 2012	N 53.62363, E 35.87014	Broad-leaved forest	Phaeozem
R2(3)	Kaluzhskiye Zaseki Nature Reserve (Southern cluster)	May 2006, July 2011, May, June, September 2012	N 53.61480, E 35.86794	Broad-leaved forest	Phaeozem
R4	Kaluzhskiye Zaseki Nature Reserve (Southern cluster)	July 2011, May, June, September 2012	N 53.62309, E 35.86900	Broad-leaved forest	Luvisol sod-podzolic
R5	Kaluzhskiye Zaseki Nature Reserve (Southern cluster)	May, September 2012	N 53.61943, E 35.87607	Young birch forest	Luvisol sod-podzolic (with arable layer)
R6	Kaluzhskiye Zaseki Nature Reserve (Southern cluster)	May, June, September 2012	N 53.63121, E 35.88146	Young birch and willow forest	Luvisol sod-podzolic
